# Dynamic Changes in the Crop Milk and Salivary Microbiota of Breeding Pigeons During the Raising Brooding Period

**DOI:** 10.3390/ani15192772

**Published:** 2025-09-23

**Authors:** Weiqing Ma, Liu Yang, Yadi Jing, Qianyuan Mo, Qingsheng Song, Changfa Wang, Mingxia Zhu

**Affiliations:** 1 College of Agriculture and Biology, Liaocheng University, Liaocheng 252000, China; mawq1994@163.com (W.M.);; 2 Agricultural Products Processing Research Institute, Chinese Academy of Tropical Agricultural Sciences, Zhanjiang 524000, China; 3 Liaocheng Huabao Poultry and Livestock Breeding Co., Ltd., Liaocheng 252000, China

**Keywords:** crop milk, saliva, microbiome, *Lactobacillus*

## Abstract

The microbiota within crop milk not only participates in the physiological regulation of parent pigeons but also promotes the healthy growth of squabs. Consequently, microbial sequencing of both crop milk and the saliva through which it passes is essential to elucidate microbial changes in both compartments during the raising brooding period. This study sampled crop milk and saliva of Mimas at three distinct time points corresponding to Days 1, 4, and 7 of raising brooding, and collected samples from three pairs of healthy breeding pigeons with similar body weights at each time point. The results demonstrated that α diversity increased in both the crop milk and salivary microbiota over time, surpassing the levels observed at the initial secretion stage. Compared to R1, the Simpson index of the salivary microbiome was significantly higher at R7 (*p* < 0.05). *Ligilactobacillus* constituted the most abundant genus in crop milk, whereas saliva harbored a greater diversity of potentially pathogenic bacteria. Phylogenetic analysis revealed a close evolutionary relationship between *Ligilactobacillus* and *Enterococcus* in crop milk, suggesting potential functional synergy. Furthermore, functional prediction indicated that ABC transporter-related genes presented the lowest expression in the crop milk microbiota during its peak secretion period. Notably, Pearson correlation analysis revealed a significant negative correlation between *Ligilactobacillus* abundance in crop milk and *Psittacicella* abundance in saliva. In summary, the crop milk and salivary microbiota exhibit distinct temporal dynamics, and their specific microbial compositions and functions can provide new research directions for the healthy breeding of squabs.

## Simple Summary

As an important poultry species and model bird, pigeons rely on crop milk as the sole source of nutrition for squabs during early development, while saliva plays a crucial role in parental feeding behaviors such as regurgitation. However, the current understanding of the composition, origin, succession patterns, and potential functional roles of the microbial communities in pigeon crop milk and saliva throughout the reproductive cycle (particularly during the brooding period) remains limited. This study tracked the dynamic changes in the microbial communities of crop milk and saliva during the brooding period, revealing their temporal succession patterns. Our findings provide essential baseline data for understanding the potential roles of crop milk microbiota in the colonization of squab gut microbiota, development of the immune system, and nutrient digestion/absorption. Furthermore, these results may offer new insights for poultry health management and probiotic development.

## 1. Introduction

Similarly to mammals, the production of crop milk in pigeons was also regulated by prolactin [1]. Moreover, pigeon milk was rich in nutrients, including protein (60%), fat (32–36%), carbohydrates (1–3%), minerals, IgA antibodies, and probiotics [2,3,4]. In mammals, milk-derived probiotics contribute to the development of the immune system in offspring. As demonstrated in piglet models challenged with cholera toxin and tetanus toxoid, breastfeeding enhances both humoral and cellular immune responses in piglets [5]. Similarly, newly hatched squabs cannot feed independently and require parental pigeons to provide nourishment through lactation, thereby obtaining essential nutrients and enhancing immunity to improve survival rates [4]. During the transfer of crop milk from parents to offspring, it passes through the crop and oral cavity. This process introduces microbiota from the crop and oral cavity into the squab’s system, facilitating the establishment of a microbial community similar to that of the parents [6]. Carbohydrates in the crop milk interact with *Bacteroides fragilis* to produce short-chain fatty acids, which help prevent necrotizing enterocolitis [7]. This interaction highlights the critical role of the microbiota in maintaining health and physiological functions. Although microbes vary across different hosts, their functions are largely mediated by conserved microbial metabolites [8]. As a specialized avian organ, the crop not only stores food but also enables preliminary fermentation [9]. The crop microbiota is dominated by lactic acid bacteria, and supplementation with exogenous lactic acid bacteria can further promote their colonization [10]. Thus, for artificially reared squabs, attention should be given not only to nutritional supplementation, but also to the composition of their microbiota.

The salivary microbiota is one of the most complex microbial communities in the human body [11]. Generally, research on the salivary microbiota has focused more on oral diseases. However, in addition to participating in the first line of defense against potential pathogens, the salivary microbiota can also serve as a diagnostic tool for detecting early physiological responses. Compared with nonpregnant women, healthy pregnant women present higher levels of *Bifidobacterium* in their saliva [12]. A Japanese study on parent–child saliva exposure revealed that infant exposure to saliva may reduce allergic reactions in preschool children [13]. The salivary microbiota can help induce immune responses in young offspring, and supplementation with *Lactobacillus reuteri* in saliva has been shown to increase IgA levels [14].

While animal genomes remain largely static, microbial communities within animals are in a constant state of dynamic flux. These changes include the establishment of early microbial communities, shifts in microbial diversity under various disease states, and microbial responses to dietary changes. By monitoring these dynamic shifts, we can identify abnormal microbial alterations and thereby maintain microbial homeostasis. Research comparing healthy and diarrheic horses revealed four fungal genera whose abundance significantly increased during bouts of diarrhea, shedding light on the critical role of gut fungal communities in equine health [15]. In broilers, the gut microbiota continuously evolves throughout their growth cycle. Within the first three days post-hatching, the gut microbiota exhibited a distinct structure, and during the rapid growth phase, significant microbial succession occurred. These findings offer insights for modulating microbial communities at different developmental stages and enhancing broiler production performance [16,17]. Studies on preterm infants have revealed that *Exiguobacterium*, *Acinetobacter*, and *Citrobacter* decline with age, whereas *Enterococcus* gradually proliferates to become dominant. *Bifidobacterial* colonization occurs later. These studies deepen our understanding of bacterial colonization in preterm infants and provide new perspectives for targeted bacterial supplementation in newborns [18]. Similarly, studies on perinatal sows have demonstrated that gut microbial functional enrichment peaks on Day 3 of lactation, indicating heightened microbial involvement in shaping the gut microbiota of suckling piglets [19].

In summary, host physiology and immune function were closely linked to microorganisms. The host shaped its microbial community through environmental selection, and these microbes in turn interacted with the host to influence metabolic and immune responses [20]. However, microbial composition was highly dynamic and subject to daily variation, regulated by factors such as fluid flow, microbial growth, and biomass [21]. In a mouse model of LPS-induced sepsis, changes in the colonic microbiota at the 12 h time point were primarily influenced by colonic contents, while by 48 h, the colonic microbial community was predominantly derived from lung tissue [22]. While the relationship between microbial dynamics and growth has been elucidated in multiple animal species, research on the dynamic changes in crop milk and salivary microbiota in pigeons is scarce. Therefore, the purpose of this study was to investigate the evolutionary changes in microbial communities in crop milk and saliva during the brooding period in squabs, and to provide a scientific basis for the healthy breeding of squabs.

## 2. Materials and Methods

### 2.1. Animals and Sample Collection

Pigeons of the Mimas breed were housed at Liaocheng Huabao Poultry and Livestock Breeding Co., Ltd. (Liaocheng, China) Each breeding pair and their offspring were maintained in individual cages under a 16 h light/dark cycle, with ad libitum access to standardized feed and water. Squabs were fed primarily by parental pigeons from Days 1–7 post-hatching, whereas crop milk synthesis peaked between Days 3–5 of the brooding period. Therefore, we collected parental saliva and crop milk samples at 3 strategic time points (Days 1, 4, and 7 of raising brooding, abbreviated as R1, R4, and R7, respectively) to capture critical phases of lactation dynamics. Specifically, at each time point, 3 breeding pairs (6 pigeons) of comparable body weights were selected. The oral cavities were gently opened, and saliva was collected with sterile rayon swabs. The swabs were immediately transferred to prelabeled 5 mL cryovials and flash-frozen in liquid nitrogen. The pigeons were subsequently euthanized via jugular exsanguination. The crop sac was aseptically dissected, and crop milk was harvested with sterile micro forceps into 2 mL cryovials for immediate immersion in liquid nitrogen. Finally, all the samples were transferred from liquid nitrogen to −80 °C ultra-low temperature freezers for 16S rRNA amplicon sequencing and metagenomic analysis. The entire protocol strictly adhered to the regulations established by the Liaocheng University Animal Welfare Committee (AP2025080401).

### 2.2. The 16S rRNA Extraction

Total genomic DNA from crop milk and the salivary microbiota was extracted using the E.Z.N.A. Soil DNA Kit (Omega Bio-tek, Norcross, GA, USA), according to the manufacturer’s protocol. The DNA concentration and purity were quantified with a NanoDrop 2000 spectrophotometer (Thermo Scientific, Wilmington, DE, USA). The integrity of the extracted DNA was verified by 1% agarose gel electrophoresis.

### 2.3. The 16S rRNA Sequencing

Using extracted DNA as a template, the V3-V4 hypervariable regions of the 16S rRNA gene were amplified via PCR with the universal primers 338F (5′-ACTCCTACGGGAGGCAGCAG-3′) and 806R (5′-GGACTACHVGGGTWTCTAAT-3′). The PCR reaction mixture comprised the following components: 4 μL of 5× TransStart FastPfu Buffer (Accurate Biology Co., Ltd., Changsha, China), 2 μL of 2.5 mM dNTPs, 0.8 μL of forward primer (5 μM), 0.8 μL of reverse primer (5 μM), 0.4 μL of TransStart FastPfu DNA Polymerase (Accurate Biology Co., Ltd., Changsha, China), and 10 ng of template DNA, with nuclease-free water added to bring the final volume to 20 μL. Amplification was performed on an ABI GeneAmp^®^ 9700 thermal cycler (Applied Biosystems, Waltham, MA, USA) using the following protocol: initial denaturation at 95 °C for 3 min; 27 cycles of amplification; and a final extension at 72 °C for 10 min.

### 2.4. High-Throughput Data Processing

Fastp (version 0.19.6) and FLASH (version 1.2.11) software were used to perform quality control and splicing on the double-ended raw sequences [23,24]. In UPARSE (version 11.0.667) software, OTU clustering was performed on the quality control concatenated sequence on the basis of 97% similarity, and chimeras were removed. The average sequence coverage of each sample reached 99.09% [25]. Using the RDP classifier, OTU species taxonomy annotation was performed on the SILVA 16S rRNA gene database, and the community composition of each sample was statistically analyzed at different species classification levels [26]. The 16S functional prediction analysis was performed with PICRUSt2 (version 2.2.0) software [27].

### 2.5. Data Analysis

Mothur (version 1.30.2) software was used to calculate the Simpson index of alpha diversity [28], PCoA based on the Bray–Curtis distance algorithm was used to test the similarity of microbial community structure between samples, and the Kruskal-Wallis H test was used to analyze whether the differences in the microbial community structures between sample groups were significant. Pearson correlation analysis was performed between the crop milk and salivary microbiota. *p* < 0.05 indicates a significant difference, and *p* < 0.01 indicates an extremely significant difference.

## 3. Results

### 3.1. Microbial Diversity Analysis of Crop Milk and Saliva

**Figure 1:**
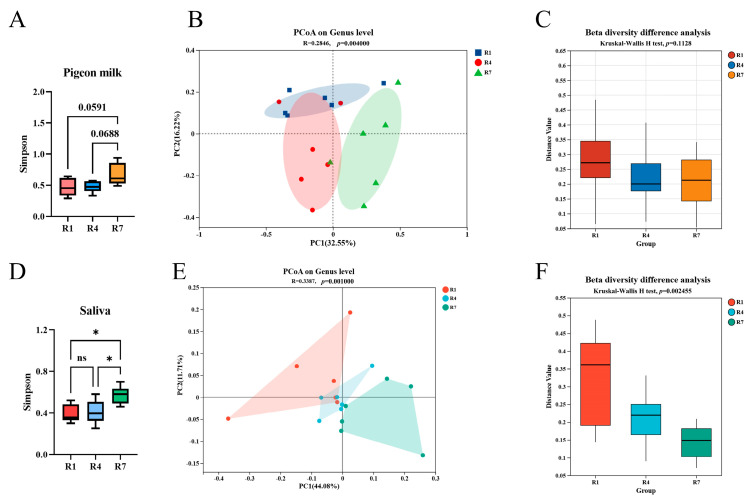
Microbial α and β diversity in crop milk and saliva. (**A**) Simpson index of crop milk mi-croorganisms. (**B**) Crop milk microbiota PcoA, the shaded area represents the confidence ellipse. (**C**) Analysis of microbial β diversity in crop milk. (**D**) Simpson index of salivary microbiota, “*” indicates *p* < 0.05, “ns” indicates *p* > 0.05. (**E**) Salivary microbiota PCoA, the shaded area represents the connection between grouped samples. (**F**) Analysis of β diversity in the salivary microbiota.

As the synthesis and secretion of crop milk by parental pigeons intensified during the brooding period, corresponding shifts occurred in the microbial communities colonizing both crop milk and saliva. Figure 1A shows that the α diversity of crop milk tended to increase at R7, but the difference was not significant. In terms of β diversity, R7 was significantly different from the other two time points but was similar in alpha diversity, as shown in Figure 1B,C. Salivary microbiota alpha diversity was significantly greater in R7 than in R1 and R4, as shown in Figure 1D. The PCoA results revealed that R7 was significantly separated from the other two groups, and the difference in beta diversity was highly significant (*p* < 0.01; Figure 1E,F).

### 3.2. Microbial Composition of Crop Milk and Saliva

**Figure 2:**
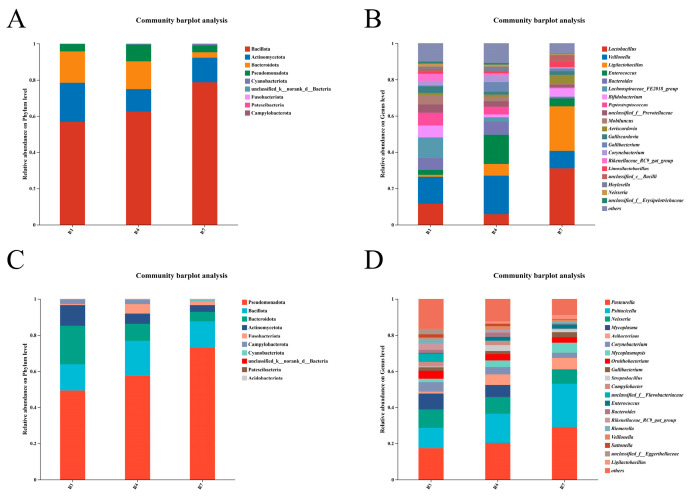
Microbial composition of crop milk and saliva. (**A**) Microbial composition of crop milk at the phylum level. (**B**) Microbial composition of crop milk at the genus level. (**C**) Microbial composition of saliva at the phylum level. (**D**) Microbial composition of saliva at the genus level.

To further understand the diversity of microorganisms in crop milk and saliva, we analyzed the microbial composition of both. At the phylum level, the crop milk microbiota is mainly composed of Bacillota, Actinomycota, Bacteroidota, and Pseudomonas, whereas at the genus level, it is mainly composed of *Lactobacillus*, *Veillonella*, and *Ligilactobacillus*, with different dominant strains at different stages, as shown in Figure 2A,B. The salivary microbiota shows a relatively regular change at the phylum level, with Pseudomonas abundance gradually increasing, whereas Bacteroidota abundance gradually decreases, as shown in Figure 2C. At the genus level, the composition of the salivary microbiota was relatively stable at different time points, with *Pasteurella*, *Psittacilla*, and *Neisseria* being the three most abundant genera, as shown in Figure 2D.

### 3.3. Microbial Differences Between Crop Milk and Saliva

**Figure 3:**
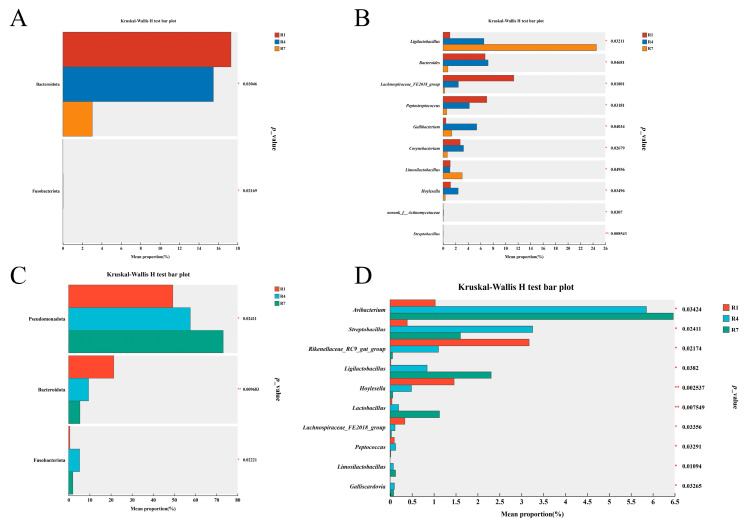
Microbial differences between crop milk and saliva. (**A**) Microbial differences in crop milk at the phylum level. (**B**) Microbial differences in crop milk at the genus level. (**C**) Microbial differences in saliva at the phylum level. (**D**) Microbial differences in saliva at the genus level. “*” indicates *p* < 0.05, “**” indicates *p* < 0.01.

Differential microorganisms are specific microbial species with significant differences in abundance between different conditions, states, or populations. To reveal the potential key microorganisms of pigeons at different time points, we conducted multiple comparative analyses of microorganisms in crop milk and saliva. The results revealed significant differences in the abundance of crop milk microorganisms between Bacteroidota and Fusobacteria at the phylum level. At the genus level, the abundance of *Ligullaceae* increased significantly, whereas the abundances of *Lachnospiraceae_FE2018_group* and *Peptostreptococcus* decreased significantly, as shown in Figure 3A,B. In terms of salivary microbiota, there were significant differences in the abundances of Pseudomonas, Bacteroidota, and Fusobacteria. At the genus level, the abundances of *Avibacterium*, *Ligullaceae*, *Lactobacillus*, and *Limosilactobacillus* increased significantly with time, whereas the abundances of *Rikenellaceae_RC9_gut_group*, *Hoylesella*, and *Lachnospiraceae_FE2018_group* significantly decreased over time, as shown in Figure 3C,D.

### 3.4. Evolutionary Analysis of Microbes in Crop Milk and Saliva

**Figure 4:**
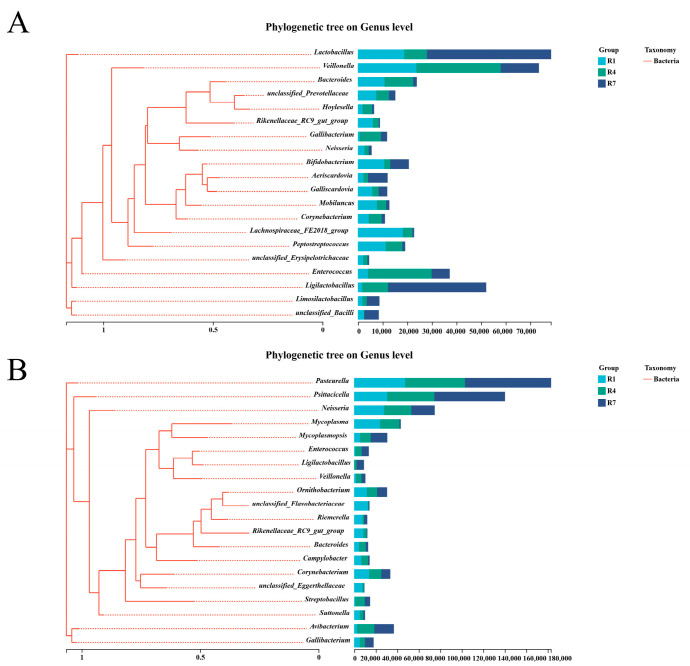
Microbial evolution analysis of crop milk and saliva. (**A**) Evolutionary analysis of crop milk microbiota. (**B**) Evolutionary analysis of salivary microbiota.

The phylogenetic relationships among microorganisms provided critical insights into their functional and taxonomic characteristics. Consequently, we constructed phylogenetic trees at the genus level for the crop milk and salivary microbiota. Figure 4A shows that among the differential microorganisms in crop milk, *Ligilactobacillus* is closely related to *Enterococcus*, whereas *Lachnospiraceae_FE2018_group* is closely related to *Peptostreptococcus*. Among the differential microorganisms in saliva, *Avibacterium* is closely related to *Gallibacter*, and more distantly related to *Ligilactobacillus* and *Rikenellaceae_RC9_gut_group*, as shown in Figure 4B.

### 3.5. Prediction of Microbial Function in Crop Milk and Saliva

**Figure 5:**
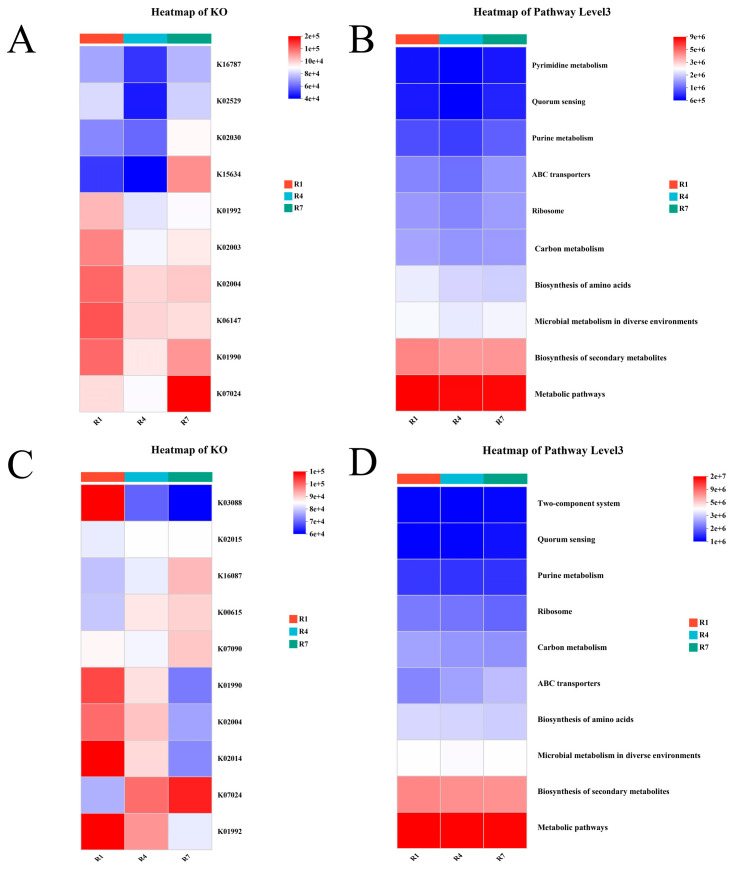
Prediction of microbial function in crop milk and saliva. (**A**) Prediction of the KO function of the crop milk microbiota. (**B**) Prediction of level 3 function of the crop milk microbiota pathway. (**C**) Prediction of the KO function of the salivary microbiota. (**D**) Prediction of level 3 function of the salivary microbiota pathway.

Through functional prediction of the KO and pathway level 3 microbial abundances, we found that the ATP-binding cassette and subfamily B bacteria of crop milk microorganisms increased or decreased with time, whereas the energy coupling factor transport system ATP-binding protein, polar amino acid transport system substrate-binding protein, and ABC-2-type transport system transmembrane protein were expressed at the lowest levels at R4. Similarly, at level 3, ABC transporters also presented the lowest expression at R4, as shown in Figure 5A,B. Unlike the crop milk microbiota, the salivary microbiota presented increases in hemoglobin/transferrin/lactoferrin receptor protein, transketolase, and sucrose-6-phosphatase levels over time. In addition, ABC transporters increased over time at level 3, as shown in Figure 5C,D.

### 3.6. Correlation Analysis of Microbes in Crop Milk and Saliva

**Figure 6:**
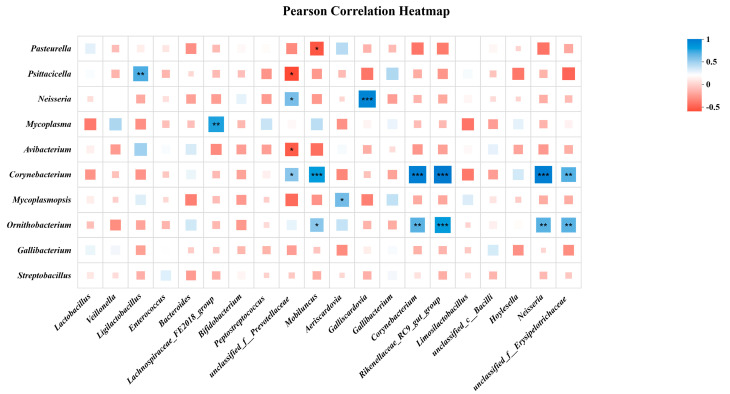
Pearson correlation analysis of microbes in crop milk and saliva. “*” indicates *p* < 0.05, “**” indicates *p* < 0.01, “***” indicates *p* < 0.001.

Finally, to understand the interaction between the crop milk microbiota and salivary microbiota, Pearson correlation analysis was conducted. It was found that the *Lactobacillus* population in crop milk was significantly negatively correlated with the *Psittacilla* population in saliva, whereas the *Lachnospiraceae_FE2018_group* in crop milk was significantly negatively correlated with the *Mycoplasma* population in saliva (Figure 6).

## 4. Discussion

The dynamic developmental patterns of the gut microbiota in pigeons have been previously documented [29]. In addition, under a constant diet, the gut microbiota of pigeons showed seasonal variations due to the influence of temperature [30]. However, research on the community succession of the crop milk and salivary microbiota remains limited. In the present study, we observed increasing trends in the microbial diversity of both crop milk and saliva over time. Although crop milk is delivered to squabs orally by parent pigeons, the temporal dynamics of the crop milk microbiota differ from those of the salivary microbiota. At the genus level, *Ligilactobacillus* was the most abundant taxon in crop milk, while *Avibacterium* contributed most significantly to the salivary microbiota. Furthermore, our results revealed a significant negative correlation between *Ligilactobacillus* abundance in crop milk and *Psittacicella* abundance in saliva, indicating that the crop milk and salivary microbiota are not independent, but exhibit interactive relationships involving competition and symbiosis.

Microbial homeostasis within animals is influenced by various environmental factors, including nutrition, immunity, and pH. However, under the influence of these factors, microbial communities undergo dynamic succession to adapt to changing environmental conditions [31]. α and β diversity, as metrics for describing microbial diversity, are crucial for elucidating the mechanisms underlying microbial environmental adaptation. Research has indicated that dietary diversity is positively correlated with β diversity, but is not associated with α diversity [14]. In contrast, this study demonstrated that the α diversity of the crop milk and salivary microbiota increased significantly over time, while β diversity also showed significant separation. These findings collectively indicate that host development and growth drive the physiological regulation of the microbiota, enabling its adaptation to the changing host environment.

Compositional analysis of the microbiota revealed that crop milk and saliva share core bacterial phyla (e.g., Bacillota and Bacteroidota), but present distinct profiles at the genus level. *Ligilactobacillus* was the most abundant genus in crop milk, which is likely associated with its role in promoting the digestion and absorption of nutrients. Consistent with previous findings, *Ligilactobacillus* also functions as a dominant genus within the pigeon gut microbiota [32,33]. As a probiotic, it contributes to maintaining gut microbial homeostasis, enhancing immune function, facilitating nutrient absorption, and alleviating inflammation to improve intestinal health [34,35]. Furthermore, *Ligilactobacillus* aids squabs in adapting to the high-protein, high-fat composition of crop milk, thereby promoting its rapid absorption [29].

Notably, differential microbial analysis revealed that brood-rearing duration modulates key functional bacterial taxa. The significant increase in *Ligilactobacillus* abundance within crop milk likely optimizes its probiotic functions for squab intestinal health. Conversely, the relative abundance of *Rikenellaceae_RC9_gut_group* in saliva exhibited a significant decrease over time. As a microbial group contributing to nutrient digestion and absorption in squabs, *Rikenellaceae_RC9_gut_group* modulates lipid metabolism via its derivative metabolite acetate [36]. This observed decline may be associated with temporal changes in the lipid and protein content of crop milk.

Phylogenetic trees depict evolutionary relationships among microorganisms, providing crucial insights into their potential functions [37]. The phylogenetic proximity between *Ligilactobacillus* and *Enterococcus* in crop milk may underpin a potential symbiotic relationship. The significant negative correlation observed between crop milk *Ligilactobacillus* and salivary *Psittacicella*, alongside the significant positive correlation between salivary *Avibacterium* and crop milk *unclassified_f__Prevotellaceae*, could be explained by the nature of *Avibacterium*. This bacterial pathogen is a nutrient-dependent commensal bacterium that specifically requires sufficient levels of the accessory growth factor nicotinamide adenine dinucleotide for proliferation in vivo [38]. Under homeostatic conditions, commensal bacteria exclude exogenous microorganisms and directly suppress pathogen growth [39].

Furthermore, functional prediction analyses corroborated the phylogenetic classification. Functional annotation revealed a downregulation of genes associated with nutrient transport, such as ABC transporters, in the crop milk microbiota during the R4 time point. This downregulation likely corresponds to specific nutritional allocation demands at this developmental stage. Conversely, the sustained upregulation of ABC transporters, transketolase, and hemoglobin receptors in the salivary microbiota aligns with functional adaptations to the dynamic oral environment during brood-rearing, enhancing nutrient acquisition and absorption capabilities.

Although the majority of microbiota transmitted via parental feeding stabilizes over time as squabs mature, the beneficial bacterial communities remain inextricably linked to facilitating digestion and absorption, and establishing the immune system during squab development [40]. This study revealed a significant negative correlation between *Ligilactobacillus* in crop milk and *Psittacicella* in saliva, suggesting that passage through the oral cavity enables crop milk to suppress harmful bacteria that is present in saliva. Pathogenic bacteria still pose a potential threat to the intestinal health of chickens, although the presence of probiotics such as Lactobacillus may inhibit these pathogens. Therefore, further investigation into the crop milk and salivary microbiota is warranted to explore strategies for enhancing squab growth efficiency, suppressing pathogenic bacteria, and reducing production losses.

## 5. Conclusions

These findings demonstrate that the α diversity of both the crop milk and salivary microbiota increased throughout the brood-rearing period, indicating dynamic compositional shifts in both communities. *Ligilactobacillus* represented the most abundant genus in the crop milk microbiota, whereas the salivary microbiota harbored a greater diversity of potentially pathogenic bacteria. Critically, we observed a significant negative correlation between *Ligilactobacillus* abundance in crop milk and *Psittacicella* abundance in saliva. The results provide a new research basis for the healthy breeding of squabs.

## Data Availability

The original contributions presented in this study are included in the article. Further inquiries can be directed to the corresponding author.
